# Glioblastoma Relapses Show Increased Markers of Vulnerability to Ferroptosis

**DOI:** 10.3389/fonc.2022.841418

**Published:** 2022-04-21

**Authors:** Helena Kram, Georg Prokop, Bernhard Haller, Jens Gempt, Yang Wu, Friederike Schmidt-Graf, Jürgen Schlegel, Marcus Conrad, Friederike Liesche-Starnecker

**Affiliations:** ^1^ Department of Neuropathology, Institute of Pathology, School of Medicine, Technical University of Munich, Munich, Germany; ^2^ Institute of AI and Informatics in Medicine, School of Medicine, Technical University of Munich, Munich, Germany; ^3^ Department of Neurosurgery, School of Medicine, Technical University of Munich, Munich, Germany; ^4^ Department of Neurology, School of Medicine, Technical University of Munich, Munich, Germany; ^5^ Institute of Metabolism and Cell Death, Helmholtz Zentrum München, Neuherberg, Germany; ^6^ Laboratory of Experimental Oncology, Pirogov Russian National Research Medical University, Moscow, Russia

**Keywords:** ferroptosis, glioblastoma, glioma, immunohistochemistry, protein expression, cell death, therapy resistance, relapse

## Abstract

**Background:**

Despite the availability of various therapy options and being a widely focused research area, the prognosis of glioblastoma (GBM) still remains very poor due to therapy resistance, genetic heterogeneity and a diffuse infiltration pattern. The recently described non-apoptotic form of cell death ferroptosis may, however, offer novel opportunities for targeted therapies. Hence, the aim of this study was to investigate the potential role of ferroptosis in GBM, including the impact of treatment on the expression of the two ferroptosis-associated players glutathione-peroxidase 4 (GPX4) and acyl-CoA-synthetase long-chain family number 4 (ACSL4). Furthermore, the change in expression of the recently identified ferroptosis suppressor protein 1 (FSP1) and aldehyde dehydrogenase (ALDH) 1A3 was investigated.

**Methods:**

Immunohistochemistry was performed on sample pairs of primary and relapse GBM of 24 patients who had received standard adjuvant treatment with radiochemotherapy. To identify cell types generally prone to undergo ferroptosis, co-stainings of ferroptosis susceptibility genes in combination with cell-type specific markers including glial fibrillary acidic protein (GFAP) for tumor cells and astrocytes, as well as the ionized calcium-binding adapter molecule 1 (Iba1) for microglial cells were performed, supplemented by double stains combining GPX4 and ACSL4.

**Results:**

While the expression of GPX4 decreased significantly during tumor relapse, ACSL4 showed a significant increase. These results were confirmed by analyses of data sets of the Cancer Genome Atlas. These profound changes indicate an increased susceptibility of relapsed tumors towards oxidative stress and associated ferroptosis, a cell death modality characterized by unrestrained lipid peroxidation. Moreover, ALDH1A3 and FSP1 expression also increased in the relapses with significant results for ALDH1A3, whereas for FSP1, statistical significance was not reached. Results obtained from double staining imply that ferroptosis occurs more likely in GBM tumor cells than in microglial cells.

**Conclusion:**

Our study implies that ferroptosis takes place in GBM tumor cells. Moreover, we show that recurrent tumors have a higher vulnerability to ferroptosis. These results affirm that utilizing ferroptosis processes might be a possible novel therapy option, especially in the situation of recurrent GBM.

## Introduction

The poor overall survival (OS) of glioblastoma (GBM) patients, even after extensive therapy including neurosurgical resection followed by combined adjuvant radiation therapy and chemotherapy with temozolomide (TMZ), is attributed to its genetic heterogeneity, diffusely infiltrating growth pattern, high proliferation rate and therapy resistance (1–3). Whilst the exact mechanisms underlying the resistance to treatment remain unknown, it seems to involve radiation-resistant tumor stem cells (4–6). Until today, the methylation status of the O-6-methylguanin-DNA methyltransferase (MGMT) promoter remains one of the most significant prognostic markers (7). Current research aims at finding new therapeutic targets to improve the patients’ prognosis. In the course of this, it was shown that the activation of the iron-dependent cell death ferroptosis can drive cancer therapy by inducing cell death, which led to the hypothesis that ferroptosis may offer new therapeutic targets for difficult-to-treat entities, including GBM (8).

The induction of ferroptosis might amplify the effect of certain chemotherapeutics (9). For instance, diffuse large B cell lymphomas and renal cell carcinomas show a high susceptibility to the ferroptosis inducer erastin (10, 11). Similar promising effects were seen for the combination of erastin with TMZ in glioma and GBM cells (12, 13).

The process of ferroptosis defines an iron-dependent oxidative destruction of lipid bilayers leading to rupturing of cellular membranes and cell death (14, 15). In 2018, ferroptosis was classified as a regulated cell death modality sensitive to lipophilic antioxidant agents (16). One way to achieve this is to perturb lipid hydroperoxide detoxification systems and to trigger iron-dependent reactive oxygen species (ROS) generation (17).

The *three hallmarks of ferroptosis* include the loss of the lipid peroxide regeneration system, an increase of polyunsaturated fatty acids (PUFAs) and an increase of redox active iron (17). The key enzyme in ferroptosis is glutathione-peroxidase 4 (GPX4). When GPX4 is either inhibited or has lost its function, ROS accumulate and cause cell death (17). Especially tumor cells rely on enzymes like GPX4 to prevent oxidative stress and assure their survival by therapy resistance. A loss of GPX4 might offer a way to selectively kill therapy-resistant tumor cells and prevent relapse (18). Because of its importance in the process, GPX4 was chosen as one marker of ferroptosis in this study.

PUFAs define the second hallmark of ferroptosis because they are more susceptible for radicals or oxidation and hence, for generating ROS. They get activated and oxidized by enzymes like acyl-CoA-synthetase 4 (ACSL4) and lipid-oxygenase. Because of its important regulatory role in in the ferroptotic cell death process (19) and its sensitizing effects on (tumor) cells towards ferroptosis (20), ACSL4 was also chosen as marker for ferroptosis in this study. Moreover, an ACSL4 depletion showed inhibiting effects on GBM tumor cell growth (20). The ferroptosis suppressor protein 1 (FSP1) was recently identified to be the second mainstay in ferroptosis control and therefore included (21).

In addition to these canonical ferroptosis players, the enzyme aldehyde dehydrogenase 1A3 (ALDH1A3) was analyzed, which has been discussed as a stem cell marker in GBM (22–25). The isoform ALDH1A3 appears to be the most active one in GBM (23). Interestingly, an *in-vitro* study showed that ALDH knock-out cells were more sensitive to therapy with TMZ compared to the wildtype cells (26).

The mechanisms through which TMZ induces ROS production (13, 27) and some type of autophagic cell death – possibly ferroptosis - are still unknown (28, 29). We hypothesize that they might involve accumulating aldehydes and complex interactions between aldehyde dehydrogenase (ALDH) (26, 30), key ferroptosis player GPX4 (13, 31), and cystine/cysteine (32). A combination of TMZ and ferroptosis inducing agents thus might be a promising approach in GBM patients (12).

The aim of the present study was to analyze the expression of ferroptosis-associated proteins in GBM. To address the expression evolvement, we compared the change the change of GPX4, ACSL4, FSP1 and ALDH1A3 expression in corresponding pairs of primary and recurrent GBM. Our study thus reveals new insights into ferroptosis in context of GBM, particularly during the course of patient treatment.

## Material And Methods

### Material

24 pairs of primary and recurrent GBM (all isocitrate dehydrogenase (IDH) wildtype; median age 58 years, range 27-78 years; 17 male, 7 female) were retrieved from the archive of the Institute of Pathology of the Technical University Munich (**Table 1**). All patients had received surgery at the Department of Neurosurgery of the Klinikum rechts der Isar between 2003 and 2017. Diagnoses were confirmed and reevaluated for this study by experienced neuropathologists according to the classification of brain tumors by the World Health Organization, 2016 (33). Clinical information was gathered by searching the hospital information system. Specification of the MGMT promotor status was obtained by searching the information system of the Institute of Pathology. The MGMT promotor status had been determined by the methylation quantification of endonuclease-resistant DNA (MethyQUESD) method (34).

**Table 1:** Patient data.

Sex	Male	17	n = 24
	Female	7	
Age at first diagnosis	median	58 y	n = 24
	range	27-78 y	
PFS/time between primary tumor and relapse	median	9 mth	n = 19
	range	3-53 mth	
OS/time between first diagnosis and death/today	median	18 mth	n = 19
	range	9-71 mth	
Time between relapse diagnosis and death/today	median	11 mth	n = 19
	range	3-56 mth	
MGMT promoter status	methylated	5	n = 22
	unmethylated	17	

24 patients have been included, of which 5 were still alive at the time of this study. y, years; mth, months. PFS, progression free survival; OS, overall survival; MGMT, O-6-methylguanine-DNA methyltransferase. The MGMT promotor status of 22 patients was available. Survival data was accessible for 19 patients.

All patients had been treated following the standard Stupp scheme (6 weeks concomitant radiochemotherapy with TMZ, followed by up to six cycles of TMZ alone (2)), and availability of tissue samples of primary and recurrent tumor was given. For GPX4, ACSL4 and ALDH1A3, 24 pairs of primary and recurrent tumors were included for immunohistochemistry (IHC) and double immunofluorescence (IF) with cell-type specific markers. The expression of FSP1 was analyzed in 13 pairs. Double immunofluorescence for GPX4 plus ACSL4 was performed for 5 pairs of primary and corresponding recurrent tumors.

A data analysis with transcriptome profiling datasets from the Cancer Genome Atlas (TCGA) regarding a change of ACSL4 and GPX4 gene expression in pairs of primary and recurrent GBM was performed to verify our results. The dataset and associated clinical information were acquired from the TCGA official website (https://portal.gdc.cancer.gov). Six corresponding pairs of primary and recurrent GBM were available (TCGA-06-0210, TCGA-06-0190, TCGA-19-4065, TCGA-14-1034, TCGA-06-0125, TCGA-06-0211). For each patient, six to nine transcriptome profiling datasets including the gene quantification expression were accessible. The ACSL4 and GPX4 gene expression from each primary GBM was compared to the corresponding recurrent GBM using the natural logarithm of the gene quantification expression (ln(expression value)).

This retrospective study was approved by the local Ethics Committee of the Technical University Munich (vote number 164/19 S-SR) and conducted in accordance with the ethical standards of the 1964 Declaration of Helsinki and its later amendments.

### Immunohistochemistry

Formalin-fixed, paraffin-embedded samples were cut in 2 μm-thin sections and deparaffinized followed by epitope unmasking in pH 6.0 citrate buffer at 95°C for 30 minutes. After incubating with endogenous peroxidase, the slides were quenched with 1.5% H_2_O_2_ and blocked in a mixture of blocking buffer (1x phosphate buffered saline (Thermo Fisher Scientific, USA), 1% bovine serum albumin (Biochrom AG, Germany), 0.2% gelatin of cold-water fish skin (SIGMA-ALDRICH^®^, St. Louis), 0.1% triton X 100 (Carl Roth GmbH+Co. KG, Germany)) with 2.5% normal horse serum (Vector Laboratories, UK) before avidin (Vector Laboratories, USA) was added. Afterwards, incubation was performed with primary antibodies against ACSL4, GPX4, ALDH1A3 and FSP1 overnight at 4˚C. The used antibodies with corresponding dilution are listed in **Table 2**. The antibody diluent consisted of blocking buffer and biotin. On the next day, biotinylated secondary anti-rabbit IgG, anti-rat IgG or anti-mouse IgG antibodies, were diluted at the rate of 1:400 and incubated for 30 minutes. Afterwards, the ABC-reagent (Vector Laboratories, USA) was applied and incubated for 30 minutes. Antibody complexes were detected with 3,3’-diaminobenzidine (DAB) reagent (Vector Laboratories, USA). Finally, counterstaining with haematoxylin was performed. Positive controls (human liver tissue for ACSL4, ALDH1A3 and FSP1; human kidney tissue for GPX4) served as quality assurance.

**Table 2:** Used antibodies.

Antibody	Company	Clone	Host species	Dilution
Anti-ACSL4	Santa Cruz, USA	Monoclonal, clone IgG_2b_	mouse	IHC: 1/100 IF: 1/20
Anti-GPX4	Abcam, UK	Monoclonal, clone EPNCIR144	rabbit	IHC: 1/3000 IF: 1/1000
Anti-ALDH1A3	Thermo Fisher Scientific, USA	polyclonal	rabbit	IHC: 1/1000
Anti-FSP1	developed in house	IgG2 monoclonal antibody raised against a N-terminal peptide of hXCT, clone 3A12-1-1	rat	IHC: undiluted
2^nd^ antibody, anti-rabbit	Vector Laboratories, USA	IgG	rabbit	IHC: 1/400
2^nd^ antibody, anti-mouse	Vector Laboratories, USA	IgG	mouse	IHC: 1/400
2^nd^ antibody, anti-rat	Vector Laboratories, USA	IgG	rat	IHC: 1/400
anti-GFAP	Dako, USA	monoclonal	mouse	IF: 1/50
anti-GFAP	Dako, USA	polyclonal	rabbit	IF: 1/500
anti-Iba1	Abcam, UK	monoclonal	mouse	IF: 1/500
anti-Iba1	Wako, USA	polyclonal	rabbit	IF: 1/500
2^nd^ antibody	Invitrogen/Thermo Fisher Scientific, USA	Polyclonal, (Alexa Fluor 568/488)	mouse	IF: 1/2000
2^nd^ antibody	Invitrogen/Thermo Fisher Scientific, USA	Polyclonal, (Alexa Fluor 568/488)	rabbit	IF: 1/2000

The cytoplasmatic staining was analyzed using the immunoreactive score (IRS) established by Remmele and Stegner (35). The score is a product of the percentage of positive cells (0 = 0%, 1 = <10%, 2 = 10-50%, 3 = 51-80%, 4 = >80%) and staining intensity (0 = no staining, 1 = weak, 2 = moderate, 3 = strong positivity) allowing total values from 0 to 12. Three randomly chosen high power fields (600-fold magnification; ocular 10-fold, objective 60-fold) containing tumor core were examined and the mean was calculated.

### Immunofluorescence

To further investigate in which cells ferroptosis in principle may occur, double immunofluorescence staining with cell markers including glial fibrillary acidic protein (GFAP), which is expressed in tumor cells and astrocytes, along with ionized calcium-binding adapter molecule 1 (Iba1) for detecting microglia cells was performed (36, 37). Like for IHC, the samples were deparaffinized, unmasked, quenched and blocked. Afterwards, anti-ACSL4 and anti-GPX4, respectively, antibodies each in combination with anti-GFAP or Iba1 antibody (all diluted in blocking buffer (1x phosphate buffered saline, 2.5% donkey serum, 1% bovine serum albumin, 0.2% gelatin of cold-water fish skin, 0.1% triton X 100) were incubated overnight at 4˚C, followed by the second antibody, which incubated for 45 minutes. The used antibodies with corresponding dilution are listed in **Table 2**. Autofluorescence Quenching Kit including 4’,6-Diamidino-2-phenylindol (DAPI) (Vector Laboratories, USA) was used to reduce autofluorescence and to counterstain the nuclei. While the ferroptosis-related enzymes were stained in a green-fluorescent dye with excitation at 488 nm detectable with the fluorescein isothiocyanate (FITC) filter, the cell type-specific markers were colored in a red-fluorescent dye with absorption at 568 nm and detected with the rhodon filter. The blue-fluorescent nuclei were detected with the DAPI filter.

The same procedure was applied to the ACSL4 and GPX4 double immunofluorescence. Only the incubation time of the first antibodies was shortened to two hours to reduce background straining.

For quantification, the amount of nuclei was counted. Afterwards, a percentage of GPX4-positive (^+^) and ACSL4^+^ cells, as well as GFAP^+^ and Iba1^+^ cells was estimated. Furthermore, the number of co-expressing cells (GPX4 or ACSL4 plus GFAP or Iba1 and ACSL4 plus GPX4) was counted. Again, three high power fields (630-fold magnification; ocular 10-fold, objective 63-fold) containing tumor core were examined and the mean calculated. Following amounts were calculated: the ACSL4- and GFAP-co-stained cells divided by the amount of GFAP-positive cells (ACSL4^+^/GFAP^+^), ACSL4^+^/Iba1^+^, GPX4^+^/GFAP^+^ and GPX4^+^/Iba1^+^, as wells as GFAP^+^/ACSL4^+^, Iba1^+^/ACSL4^+^, GFAP^+^/GPX4^+^ and Iba1^+^/GPX4^+^.

### Statistical Analysis

Statistical analyses were performed with R Version 3.6.1. Since pairs of samples from the same patient had to be compared and a normal distribution was not always given, all significance was tested using the Wilcoxon signed-rank tests were used for comparisons of primary and recurrent tumors and Spearman’s rank correlation coefficients with corresponding tests for assessment of associations between quantitative data. Since the TCGA-dataset was also not coherently normally distributed, the Wilcoxon signed-rank test was applied once more. For correlating the IHC results with clinical outcome, a test on association was performed and a cut-off score was estimated using the ‘coin’ package which calculated the best threshold to discriminate patients with regard to OS (38). Kaplan-Meier survival curves are shown for the corresponding groups. Moreover, the p-value was also estimated by the “maxstat”-function from the “coin”-package. For all tests, statistical significance was defined as p<0.05.

## Results

### Dynamic Changes in Ferroptosis-Related Enzymes in Primary and Recurrent GBM

By immunohistochemistry, a significant increase of ASCL4 and ALDH1A3 expression and a significant decrease of GPX4 expression was observed when comparing primary and corresponding recurrent tumor. FSP1 expression increased slightly, not significantly, though.

Following, the results are demonstrated in detail. The IRS allows values from 0 to 12. Inconsistencies between absolute values and difference (Δ) are due to rounding.

**Figure 1:**
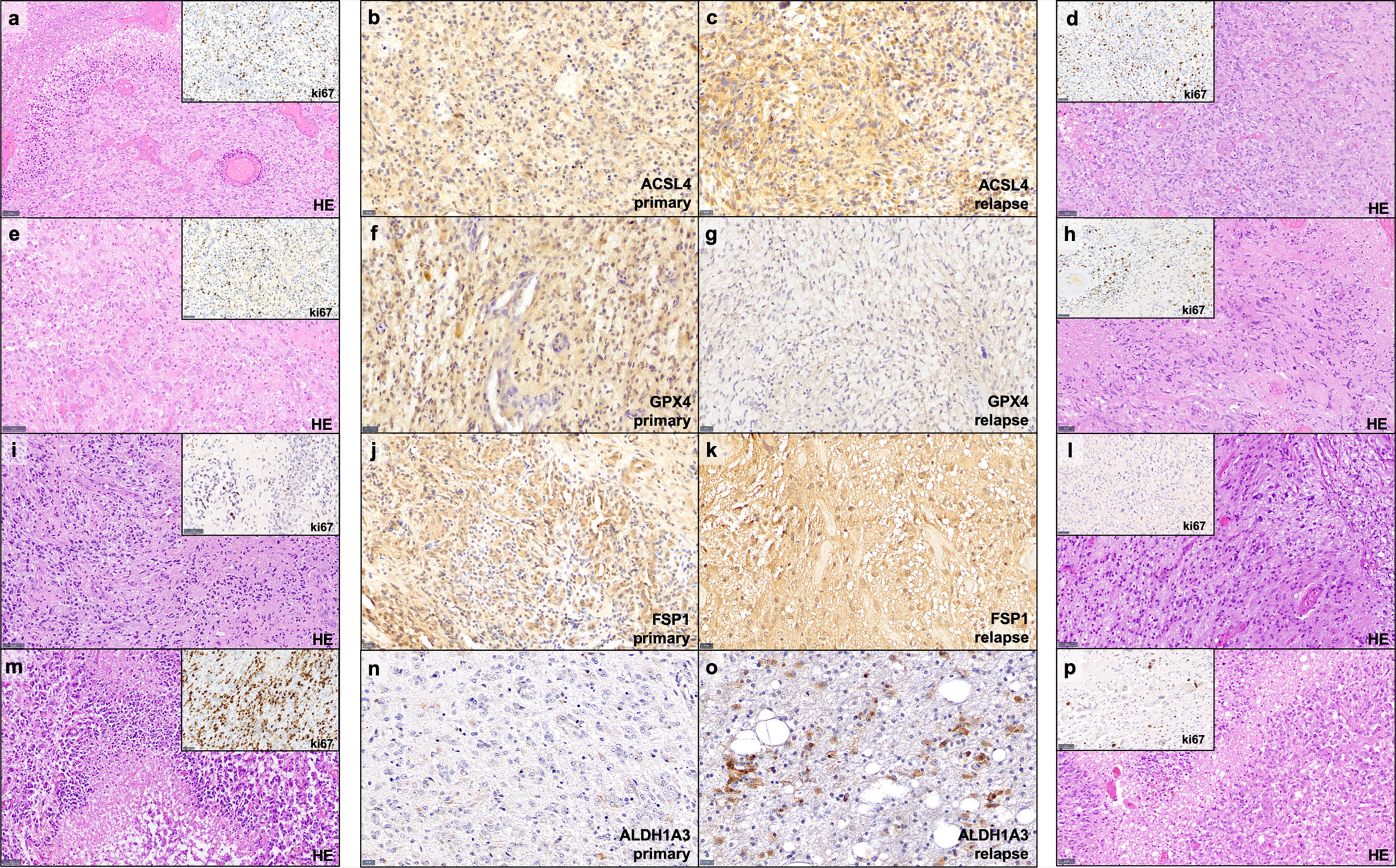
Examples of immunohistochemistry staining. Shown are paired GBM primary and relapse with stronger ACSL4 and ALDH1A3 expression in the relapse **(C, O)** compared to the primary tumor **(B, N)**. In contrast, GPX4 and FSP1 display a stronger expression in the primary tumor **(F, J)** compared to their relapse **(G, K)**. Furthermore, for each example of primary and recurrent GBM, a hematoxylin and eosin (HE) stains as well as a ki67 immunohistochemistry of representative areas are provided **(A, D, E, H, I, L, M, P)**. Scale bars of ACSL4, GPX4, FSP and ALDH1A3 immunohistochemistry: all 20 μm; Scale bars of HE and ki67 immunohistochemistry: all 50 μm.

**Figure 2:**
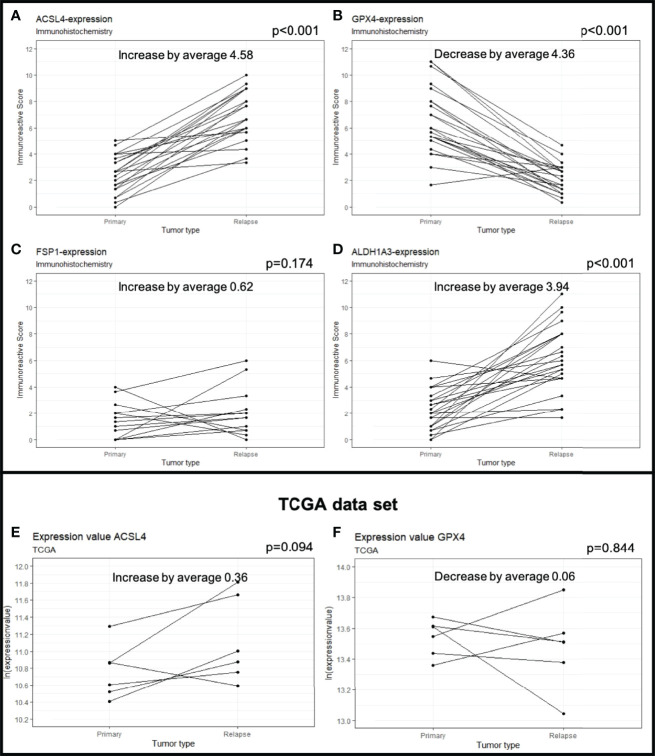
Protein expression in primary and recurrent GBM. The dots mark the level of IRS in the primary and relapse tumor. Each line represents the change in expression of one patient, showing increase of expression for ACSL4 **(A)**, FSP1 **(C)** and ALDH1A3 **(D)**. GPX4 expression decreases significantly in 23 out of 24 patients **(B)**. The results were verified by analyses of the TCGA data set showing an increase in ACSL4 expression **(E)** and a decrease in GPX4 expression **(F)**.

The average of ACSL4 expression increased from IRS 2.40 in the primary tumors to IRS 4.99 in the recurrent tumors (**Figures 1B, C**, **2A**). This change of 4.58 IRS points was highly significant (p<0.001).

Expression of GPX4 decreased from IRS 6.53 in the primary to IRS 2.17 in the recurrent tumors (Δ 4.36 IRS points, **Figures 1F, G**, **2B**). The decrease could be detected in 23 out of 24 patients and was highly significant (p<0.001).

FSP1 expression increased slightly from IRS 1.46 to 2.08 (Δ 0.62 IRS points). This change was not significant (p=0.174, **Figures 1J, K**, **2C**).

ALDH1A3 expression increased in 22 out of 24 patients (**Figures 1N, O**, **2D**). The increase from IRS 2.24 in the primary to IRS 6.18 in the recurrent tumors (Δ 3.94 IRS points) was highly significant (p<0.001).

The complete results of immunohistochemistry are summarized in **Table 3**.

**Table 3:** Summary of results of immunohistochemistry.

Protein	IRS total		IRS primary		IRS recurrent		Δ primary-recurrent					
	Mean	Range	Mean	Median	Mean	Median	Mean	Median	SD	p-value	Z-score
ACSL4	4.69	0.00-10.00	2.40	2.50	6.99	6.67	+4.58	+5.00	2.12	<0.001	-7.181
GPX4	4.35	0.33-11.00	6.53	6.00	2.17	2.17	-4.36	-4.33	2.25	<0.001	-7.047
FSP1	1.77	0.00-6.00	1.46	1.33	2.08	1.67	+0.62	+0.67	2.28	0.174	-1.360
ALDH1A3	4.21	0.00-11.00	2.24	2.00	6.18	5.83	+3.94	4.17	2.91	<0.001	-6.672

A TCGA data analysis was performed to verify our results. The gene expression level was normalized using fragments per kilobase of transcript per million mapped reads (FPKM). Although insignificant (p=0.094), the ACSL4 gene expression increased in five out of six patients (TCGA-06-0210, TCGA-06-0190, TCGA-19-4065, TCGA-06-0125, TCGA-06-0211) by an average of 0.36 (**Figure 2E**). Moreover, the GPX4 gene expression decreased in four out of six patients (TCGA-06-0210, TCGA-06-0190, TCGA-19-4065, TCGA-14-1034) by an average of 0.06 (**Figure 2F**), again, not significantly, though (p=0.844). The insignificant results may be allegeable by the small sample size.

The results of the TCGA data analysis are summarized in **Table 4**. **Figure 1** also includes hematoxylin and eosin stain, as well as ki67 immunohistochemistry as proliferation marker for all examples of primary and recurrent GBM.

**Table 4:** Summary of the TCGA analysis.

Protein	Ln(expression-value) total		Ln(expression-value) primary		Ln(expression-value) recurrent		Δ primary-recurrent					
	Mean	Range	Mean	Median	Mean	Median	Mean	Median	SD	p-value	Z-score
ACSL4	7.22	1.42-12.90	7.07	7.47	7.40	7.70	+0.36	+0.36	0.42	0.094	-1.676
GPX4	9.45	4.06-14.95	9.51	9.25	9.38	9.07	-0.06	-0.08	0.31	0.844	-0.197

### Co-Expression Analysis With Immunofluorescence

**Figure 3:**
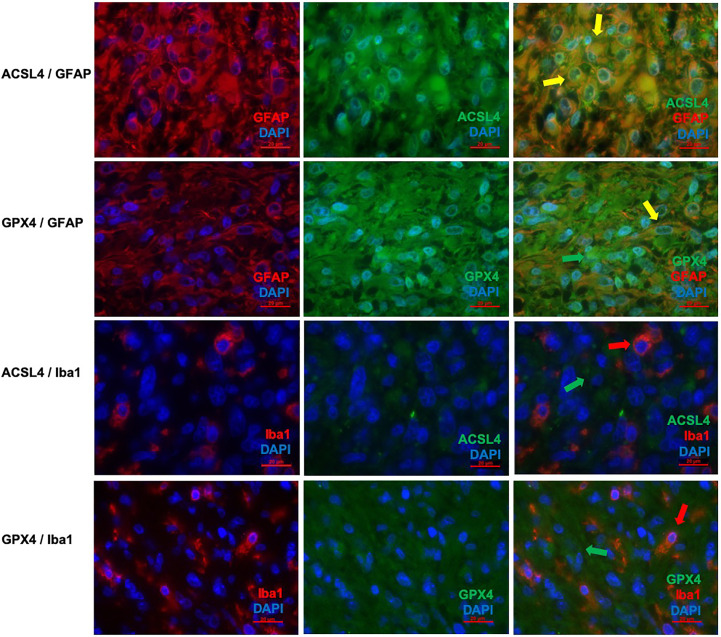
Co-staining of ferroptosis-related proteins with cell type specific markers. **(A–C)** Double immunofluorescence with ACSL4 and GFAP in a primary GBM shows several cells with co-expression (yellow arrow). **(D–F)** Double immunofluorescence with GPX4 and GFAP shows co-expression in many cells in a primary GBM (yellow arrow). Additionally, one cell expressing only GPX4 (green arrow) is marked. **(G–I)** Double immunofluorescence with ACSL4 and Iba1 in a primary GBM shows several Iba1^+^ cells which mostly do not express ACSL4 (red arrow: Iba1-positive cell lacking ACSL4 expression; green arrow: ACSL4-positive non-microglial cell). **(J–L)** Double immunofluorescence with GPX4 and Iba1 in a relapse GBM. Most cells express either GPX4 (green arrow) or Iba1 (red arrow). Scale bars all 20 μm.

Co-expression of ferroptosis-related proteins ACSL4 and GPX4 with the cell type specific markers GFAP and Iba1 was analyzed to further investigate which cell types become vulnerable to ferroptosis. Many GFAP-positive (^+^) cells expressed the ferroptosis-associated markers labelled by a yellow signal (**Figures 3A–F**). In combination with Iba1, however, only a few cells showed a clear yellow signal indicating co-expression with ACSL4 and GPX4 (**Figures 3G–L**). To anticipate: the co-expression analysis shows that 70% to 80% of the cells expressing the ferroptosis-associated marker genes ACSL4 or GPX4 do also express GFAP.

**Figure 4:**
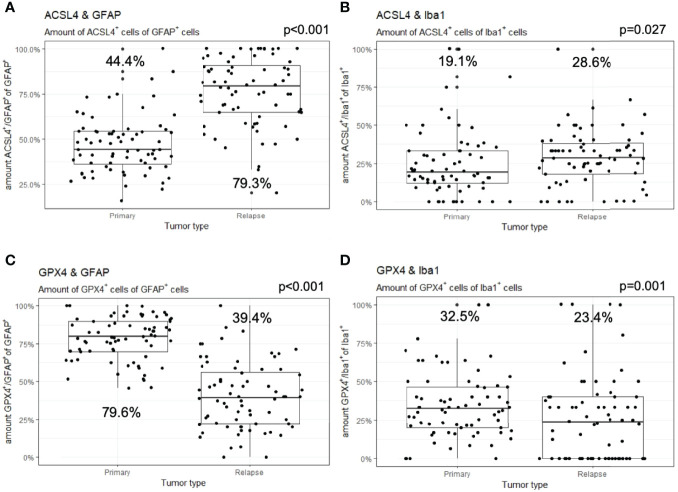
Co-expression of ferroptosis-associated markers with GFAP and Iba1. Given are the numbers of ACSL4^+^ or GPX4^+^ cells of GFAP^+^ or Iba1^+^ cells. The dots indicate the amounts of each patient in the primary and relapse tumor. **(A)** The number of ACSL4^+^/GFAP^+^ cells increases significantly in the relapse. **(B)** The amount of ACSL4^+^/Iba1^+^ cells increases significantly in the relapse. **(C)** The number of GPX4^+^/GFAP^+^ cells decreases significantly in the relapse. **(D)** The amount of GPX4^+^/Iba1^+^ cells decreases significantly in the relapse.

In detail and illustrated in **Figures 4A, B**, the overall amount of ACSL4^+^ cells in both combinations increased significantly from primary to recurrent tumors. Moreover, a higher amount of GFAP^+^ cells also expressed ACSL4 compared to Iba1^+^ cells. The amount of ACSL4^+^ cells of the GFAP^+^ cells increased significantly by an average of 29.3% (p<0.001) between primary and relapsed tumor. The number of ACSL4^+^ cells in the Iba1^+^ cell population increased significantly by an average of 4.2% (p=0.027).

The count of GPX4^+^ cells of GFAP^+^ cells decreased significantly by an average of 38.0% between primary and recurrent tumors (p<0.001), while the amount of GPX4^+^ cells of Iba1^+^ cells decreased significantly by an average of 9.2% (p=0.001) in the relapse (**Figures 4C, D**).

**Figure 5:**
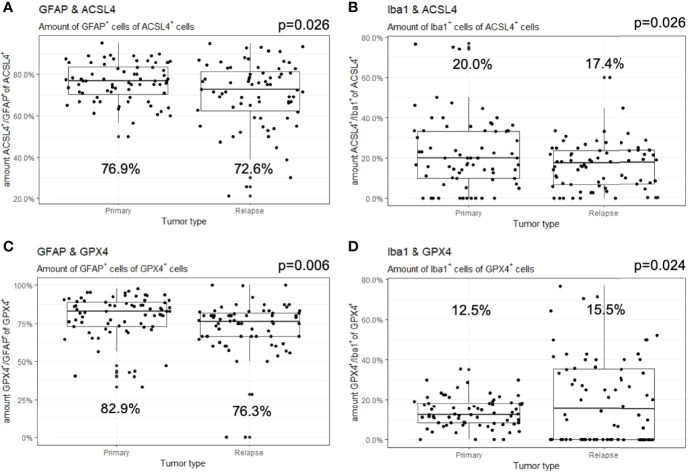
Co-expression of GFAP and Iba1 with ACSL4 and GPX4. Given are the numbers of GFAP^+^ or Iba1^+^ cells of ACSL4^+^ or GPX4^+^ cells. The dots indicate the amounts of each patient in the primary and relapse tumor. **(A)** The number of GFAP^+^/ACSL4^+^ cells decreases insignificantly in the relapse. **(B)** The amount of Iba1^+^/ACSL4^+^ cells significantly decreases in the relapse. **(C)** The number of GFAP^+^/GPX4^+^ cells decreases significantly in the relapse. **(D)** The amount of Iba1^+^/GPX4^+^cells increased significantly in the relapse.

The number of GFAP^+^ of ACSL4^+^ cells decreased insignificantly by an average of 5.8% in the recurrent tumors (p=0.026, **Figure 5A**). Moreover, the amount of Iba1^+^ cells of ACSL4^+^ cells was three to four times lower in the primary tumor and decreased significantly by an average of 6.1% in the recurrent tumor (p=0.026; **Figure 5B**).

A similar tendency can be observed in **Figure 5C** with the amount of GFAP^+^ cells of GPX4^+^ cells. This number decreased significantly by an average of 6.9% (p=0.006). On the other hand, the amount of Iba1^+^ cells of the GPX4^+^ cell population was three to four times lower (**Figure 5D**). It increased significantly by an average of 6.6% (p=0.024). The quantitative results of the described double immunofluorescences are summarized in **Table 5**.

**Table 5:** Summary of results of co-expression analysis by immunofluorescence.

Proteins		primary		recurrent		Δ primary-recurrent				
		Mean	Median	Mean	Median	Mean	Median	SD	p-value	Z-score
ACSL4^+^ in	GFAP^+^	47.1%	44.4%	76.5%	79.3%	+29.3%	+30.8%	18.6%	<0.001	-6.647
	Iba1^+^	24.6%	19.1%	28.7%	28.6%	+4.2%	10.4%	19.3%	0.027	-2.209
GPX4^+^ in	GFAP^+^	78.4%	79.6%	40.3%	39.4%	-38.0%	-40.4%	18.1%	<0.001	-7.124
	Iba1^+^	34.5%	32.5%	25.3%	23.4%	-9.2%	-9.7%	20.0%	0.001	-3.192
GFAP^+^ in	ACSL4^+^	76.1%	76.9%	70.3%	72.6%	-5.8%	-4.4%	14.7%	0.026	-2.223
Iba1^+^ in		22.2%	20.0%	16.1%	17.4%	-6.1%	-0.1%	16.0%	0.026	-2.232
GFAP^+^ in	GPX4^+^	79.1%	82.9%	72.1%	76.3%	-6.9%	-6.6%	17.1%	0.006	-2.747
Iba1^+^ in		13.6%	12.5%	20.2%	15.5%	+6.6%	+4.9	15.1%	0.024	-2.255
ACSL4^+^ with	GPX4^+^	18.9%	15.7%	16.2%	17.1%	-2.7%	-3.4%	7.5%	0.625	-7.181

**Figure 6:**
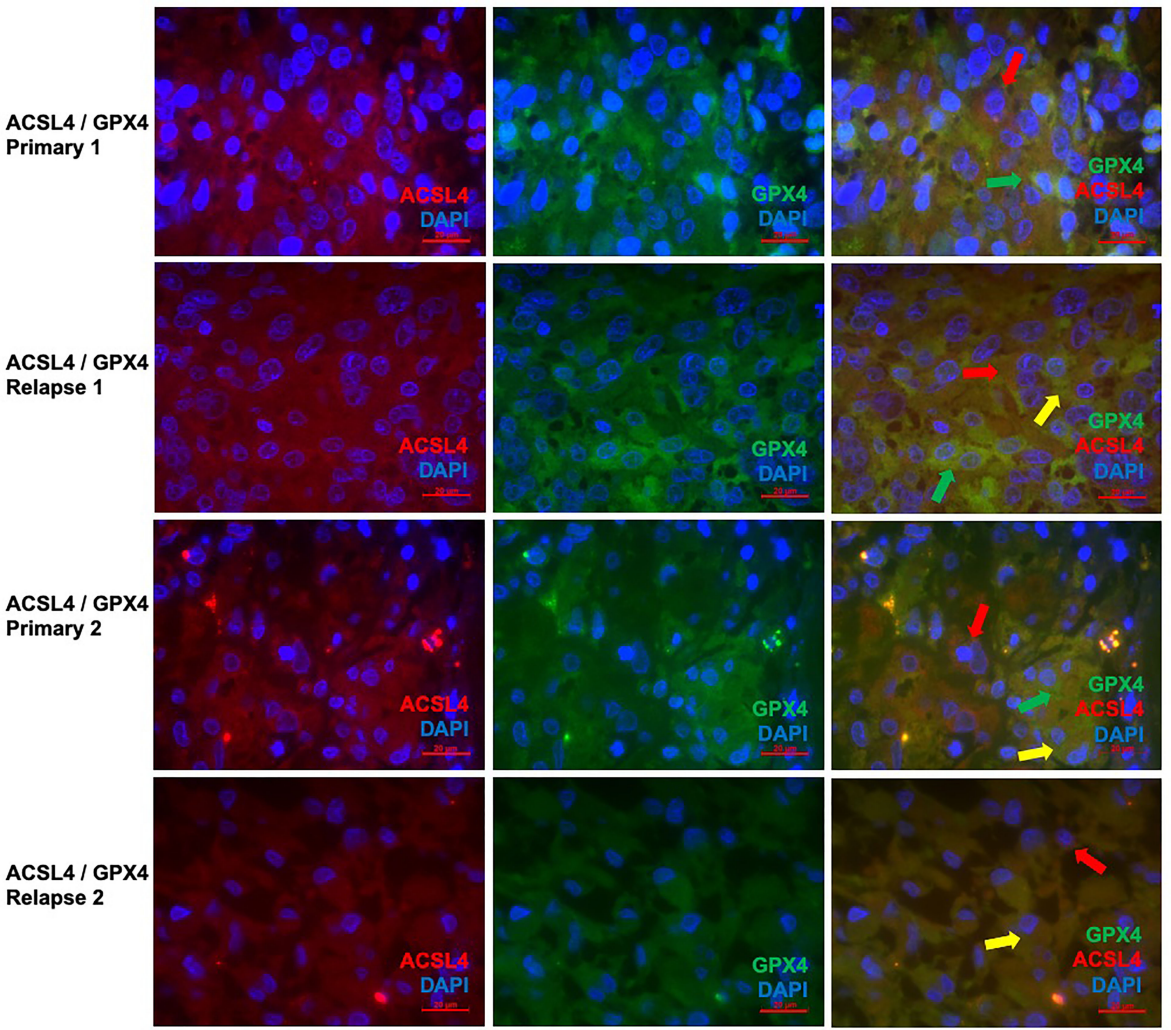
Co-staining of both ferroptosis-related proteins in pairs of primary and recurrent GBM. **(A–C)** Double immunofluorescence with ACSL4 and GPX4 in a primary GBM (patient A) shows cells expressing either ACSL4 (red arrow) or GPX4 (green arrow). **(D–F)** Double immunofluorescence with ACSL4 and GPX4 in a relapse GBM (patient A) shows several cells with co-expression (yellow arrow), as well as only ACSL4^+^ cells (red arrow) and GPX4^+^ cells (green arrow). **(G–I)** Double immunofluorescence with ACSL4 and GPX4 in a primary GBM (patient B) shows ACSL4^+^ cells (red arrows) and GPX4^+^ cells (green arrow). Some cells express both ACSL4 and GPX4 (yellow arrow). **(J–L)** Double immunofluorescence with ACSL4 and GPX4 in a relapse GBM (patient B). Whilst many cells show co-expression (yellow arrow), one cell only expressing ACSL4 is marked (red arrow). Scale bars all 20 μm.

Since the changes in expression of ACSL4 and GPX4 from primary to relapse GBM were already analyzed quantitively in the IHC, double immunofluorescence of these two proteins was performed to show parallel expression and possible interactions in a qualitatively way. With a mean of 18.9% in primary and a mean of 16.2% in relapse GBM, several cells show co-expression, although there is no significant difference (p=0.625). Whilst GPX4 remained prominent in the primary tumors (**Figures 6A–C, G–I**), ACSL4 was expressed more in relapse GBM (**Figures 6D–F, J–L**), affirming the results of IHC.

### Association of Ferroptosis-Associated Markers With Overall Survival

**Figure 7:**
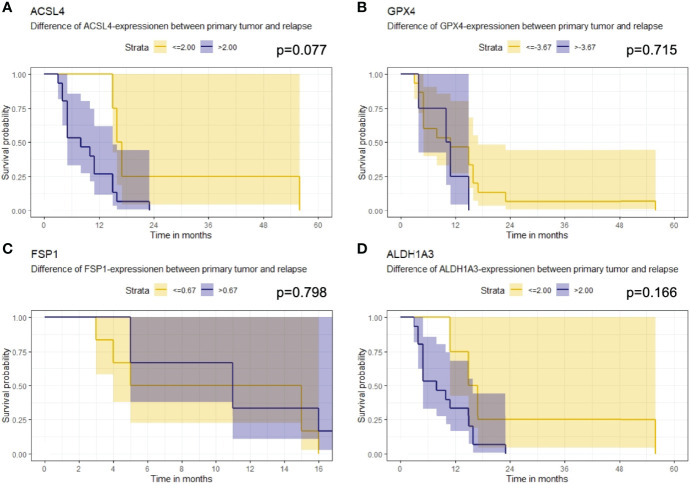
Kaplan-Meier survival curves. Starting point for measurement of survival time was the time at recurrent confirmation. The p-value describes a possible association between enzyme expression and OS. **(A)** Patients whose ACSL4 expression increased by more than 2.00 IRS points had a worse outcome, although. **(B)** GPX4 cutpoint is at -3.67. Patients with a higher pronounced decrease in GPX4 expression had a slightly better outcome. **(C)** FSP1 cutpoint is at 0.67. There is no relevant association. **(D)** ALDH1A3 cutpoint is at 2.00. Patients with a higher increase had a worse outcome.

Furthermore, the association of the expression dynamic of ACSL4, GPX4, FSP1 and ALDH1A3 with OS was evaluated. The IRS differences between primary and recurrent tumor were calculated for each marker and a threshold for every enzyme was estimated. Patients with a larger increase than 2.00 in their ACSL4 expression (**Figure 7A**) showed a poorer overall survival with a median of 8 months compared to patients with an increase of up to 2.00 and with a median OS of 16.5 months. However, no significant association between ACSL4 expression an OS was observed (p=0.077). Given that the GPX4 expression decreased in almost all patients, the calculated cutpoint amounts to -3.67 (**Figure 7B**). Patients with an even stronger decrease (≤ -3.67) displayed a slightly better overall survival with a median of 11 months compared to patients with a smaller decrease with a median of 10.5 months, yet again no statistically significant association between expression and OS was found (p=0.715).

The threshold for FSP1 was 0.67 (**Figure 7C**). Patients with a higher increase in expression had a median OS of 11.0 months, those with a lower change had a median OS of 10.0 months. There was no significant association between FSP1 expression and OS (p=0.798). For ALDH1A3 expression, a cut-off value of 2.00 was identified (**Figure 7D**). Patients with a stronger increase trended to have a poorer overall survival with a median of 8.0 months compared to the patients with less increase than 2.00 IRS points with a median OS of 16 months. Again, no significant association was observed (p=0.166).

In addition, the change in expression of each enzyme was correlated with the methylation status of the MGMT promoter. No significant correlations between the difference of expression of ACSL4 (rho=-0.146, p=0.518), GPX4 (rho=0.205, p=0.359), ALDH1A3 (rho=0.146, p=0.518) or FSP1 (rho=0.336, p=0.336) and status of methylated MGMT promoter were detected.

The results of the survival analysis are summarized in **Table 6**.

**Table 6:** Summary of results of survival analysis.

Protein	Cutpoint	Time between relapse diagnosis and death		p- value
		Mean	Median	
ACSL4	≤2.00	26.0	16.5	0.077
	>2.00	9.3	8.0
GPX4	≤-3.67	13.6	11.0	0.715
	>-3.67	10.0	10.5
FSP1	≤0.67	9.7	10.0	0.798
	>0.67	10.8	11.0
ALDH1A3	≤2.00	24.8	16.0	0.166

## Discussion

Despite extensive therapy, the prognosis of GBM remains very poor. Therefore, new therapeutic approaches are a key topic of many studies. Ferroptosis has been discussed as possible novel therapeutic target in cancer, particularly in light of a number of recent reports that suggested that therapy-resistant cancer cells and those undergoing epithelial-mesenchymal transition display a high vulnerability towards ferroptosis (8, 39). Thus, the objective of this study was to analyze whether ferroptosis is in principle activated in GBM and moreover, if there is a difference in vulnerability between primary and relapsed tumors. Various therapy options examined the effect of ferroptosis induction *in vitro* and ex vivo (17, 19, 40). All of these studies could in fact confirm an increased therapy response after the induction. Therefore, the following enzymes implicated in the process of ferroptosis were chosen (17): ACSL4, GPX4 and FSP1.

Our study shows that ACSL4 expression increases in GBM relapses compared to their primaries, whilst the GPX4 expression decreases. The results of the TCGA data analysis verified these results. An increased expression of ACSL4 is ultimately linked to an increased generation of activated PUFAs, which are used to produce oxidative stress and activate even more PUFAs by forming radicals in the presence of iron. Upon esterification into membranes they may become peroxidized thus rendering cells more sensitive to ferroptosis (19, 41, 42). Moreover, the decrease of GPX4 expression implies that the detoxifying capacity might be diminished. Both changes increase the propensity of cells to undergo lipid peroxidation (19, 43). This can also be caused by chemotherapeutics, radiation or simply burning energy (44–46).

Since FSP1 was recently shown to efficiently protect against ferroptosis caused by GPX4 deletion or inhibition (21, 47), it was hypothesized that its expression may increase in response to a loss of GPX4 expression. Accordingly, a slight, although not significant increase in expression could be detected. Unlike the glutathione/GPX4 axis that directly reduces lipid hydroperoxides in the membranes to its corresponding alcohols, the oxidoreductase FSP1 regenerates extra-mitochondrial ubiquinone to ubiquinol, that in turn either directly or indirectly *via* vitamin E prevents the lipid peroxidation chain reaction by reducing peroxyl radicals in phospholipid acyl chains (8). Furthermore, FSP1 was described as a protein prohibiting ferroptosis through the suppression of lipid peroxidation. Although two of the defined hallmarks can be detected in GBM relapse, additional studies are warranted to show that there is indeed increased lipid peroxidation in respective tissues as this would ultimately tell us that an imbalance in PUFA enrichment of membranes and a compromised protecting system sensitizes tumors to ferroptosis (17). The antibody against human FSP1 was reported in 2019 (21). Thus, its analysis was complemented retroactively with sufficient material from only 13 patients left.

This is the first study that investigated a potential relationship between ALDH1A3 and ferroptosis susceptibility. IHC demonstrated a significant increase of ALDH1A3 between GBM primary and relapse. This is in accordance with the mesenchymal transformation taking place during occurrence of recurrent tumors or a selection of GBM tumor stem cells surviving. ALDH1A3 has been associated with mesenchymal differentiation in GBM by keeping cells in an undifferentiated, stem-cell-like state which might also lead to therapy resistance (22–24). Since ALDH1A3 is involved in the detoxification of aldehydes generated as secondary products by lipid peroxidation, an increase in ALDH1A3 expression could present a cellular response towards more lipid peroxidation in GBM relapse.

Furthermore, since the quantitative changes of ACSL4 and GPX4 were already analyzed in the IHC results, the double immunofluorescence with ACSL4 and GPX4 was performed on only 5 pairs of primary and relapse GBM to demonstrate possible interactions. The detected co-expression, with GPX4 dominating in the primary and ACSL4 in the relapse GBM, indicates a complex equilibrium-like relation between the two ferroptosis-markers. Regarding this, Sha et al. examined the combined status of ACSL4 and GPX4 expression in breast cancer patients. They discovered that the combined status could predict pathological complete response to chemotherapy due to their balance-like interactions. Moreover, patients with a high ACSL4 and low GPX4 status showed higher sensitivity to chemotherapy leading to the assumption that a combination of ACSL4 inducer and GPX4 inhibitor could be beneficial for treatment efficacy (48).

In combination with the results of the double immunofluorescence with GFAP and Iba1, we furthermore provide intriguing evidence that ferroptosis is more likely to take place in GBM tumor cells and not in the surrounding microglia cells.

There was no significant association between the change of expression of ferroptosis-associated proteins and OS. Nevertheless, patients with a high increase of ACSL4 expression had a poorer OS than those with a low increase. This suggests that patients with a higher content of PUFAs in membranes have a poorer overall outcome. Liu et al. identified 19 ferroptosis-related genes in glioma using data from genome atlases including TCGA, upon which they evaluated a risk score (49). The risk score of those genes positively correlated with glioma malignancy, as well as migration and invasion. While higher risk scores regarding the ferroptosis-related genes were associated with worse prognosis, the receiver operating characteristic curve generated by the risk score could predict patient OS. Since six signature genes of the 19 ferroptosis-related genes were involved in the GPX4 regulation, GPX4 and its role in ferroptosis might play a crucial role regarding survival of glioma patients. Furthermore, mesenchymal cancer cells associated with drug resistance proofed to be selectively dependent on GPX4 (18). Therefore, GPX4 inhibitors were selectively lethal to these cells, offering yet another therapeutic option. Moreover, patients with a higher ALDH1A3 increase showed poorer OS. This might lead to the assumption that the detoxifying systems including GPX4 and ALDH1A3 can sense the oxidative stress level in the cell and therefore coordinate up- and downregulation accordingly. When there is more ROS accumulating due to the downregulation of one of the systems, the other one possibly increases.

The lack of significance may be attributable to the small sample size and, therefore, to the wider confidence intervals. Nevertheless, these results support the hypothesis that an increased generation of lipid hydroperoxides and increased vulnerability towards ferroptosis may occur during primary and relapse diagnosis. It remains, however, to be explored, whether TMZ in fact contributes to the sensitization of GBM towards the ferroptotic process. Sehm et al. combined several ferroptosis inducers like erastin and sorafenib with TMZ and showed that TMZ works in an xc-system expression dependent manner (12). Furthermore, Buccarelli *et al.* reported increased glioblastoma stem-like cells susceptibility to TMZ after induction of ferroptosis (40). TMZ treatment thus may act as a possible ferroptosis inducer but further experiments including a control group without the treatment remain necessary. Moreover, the amount of TMZ may have an effect on this process. If more TMZ, maybe in form of more TMZ cycles, influences, or even amplifies the ferroptosis induction, has yet to be investigated.

A shortcoming of this study is the small sample size, which can be explained by the poor prognosis of GBM, where death occurs often before relapse and in case of relapse situation, only a small portion of GBM patients receive re-resection. The high value of our cohort is demonstrated by considering the small number of 6 pairs of primary and recurrent GBM available at the TCGA data base. The heterogeneity of GBM was not fully respected in this study. By calculating a mean of three randomly chosen tumor containing areas, we tried to incorporate the heterogeneity, though. As the heterogeneity plays a key role for therapy resistance, following studies should address differences in intratumoral expression. Another limitation consists of a missing control group.

To conclude, this is the first study analyzing ferroptotic processes in GBM between the primary and relapse tumor. Based on our results, ferroptosis likely takes place in GBM tumor cells. Moreover, we showed that there is a dynamic in the expression of ferroptosis-associated between primary and recurrent GBM with a higher vulnerability to ferroptosis in the relapses. These results affirm that utilizing ferroptosis processes might be a possible novel therapy option especially in the situation of recurrent GBM. Particularly relevant for GBM is the role of TMZ, although it remains to be determined whether it acts as a true ferroptosis trigger or a sensitizer. Nonetheless, prospective trials should be geared to examine a possible link between TMZ and ferroptosis and to validate its true clinical value.

## Conclusion

Our study implies that ferroptosis may take place in GBM tumor cells due to the profound changes in the expression of ACSL4 and GPX4. Moreover, we show that recurrent tumors have a higher vulnerability to ferroptosis. These results affirm that utilizing ferroptosis processes might be a possible novel therapy option especially in the situation of recurrent GBM.

## Data Availability Statement

The raw data supporting the conclusions of this article will be made available by the authors, without undue reservation.

## Ethics Statement

The studies involving human participants were reviewed and approved by Ethics committee of the Technical University Munich. Written informed consent for participation was not required for this study in accordance with the national legislation and the institutional requirements.

## Author Contributions

HK, MC, JS, YW, and FL-S contributed to conception and design of the study. HK, JG, GP, and FS-G organized the database. HK and BH performed the statistical analysis. GP performed the TCGA analyses. HK wrote the first draft of the manuscript. MC, JS, and FL-S wrote sections of the manuscript. All authors contributed to manuscript revision, read, and approved the submitted version.

## Funding

Work in the Conrad lab is supported by the Deutsche Forschungsgemeinschaft (DFG) CO 291/7-1, the DFG Priority Program 2306 (CO 297/9-1; CO 297/10-1), and the Ministry of Science and Higher Education of the Russian Federation (075–15–2019–1933). MC is further funded by the European Research Council (ERC) under the European Union’s Horizon 2020 research and innovation programme (grant agreement No. GA 884754).

## Conflict of Interest

The authors declare that the research was conducted in the absence of any commercial or financial relationships that could be construed as a potential conflict of interest.

## Publisher’s Note

All claims expressed in this article are solely those of the authors and do not necessarily represent those of their affiliated organizations, or those of the publisher, the editors and the reviewers. Any product that may be evaluated in this article, or claim that may be made by its manufacturer, is not guaranteed or endorsed by the publisher.

## Abbreviations

^+^ = positive; ACSL4, acyl-CoA-synthetase long chain family member 4; ALDH, aldehyde dehydrogenase; DAB, 3,3’-diaminobenzidine; DAPI, 4’,6-Diamidino-2-phenylindol; FITC, fluorescein isothiocyanate; FPKM, fragments per kilobase of transcript per million mapped reads; FSP1, ferroptosis suppressor protein 1; GBM: glioblastoma; GFAP, glial fibrillary acidic protein; GPX4, glutathione peroxidase 4; HE, hematoxylin and eosin; Iba1, ionized calcium-binding adapter molecule 1; IDH, isocitrate dehydrogenase; IHC, immunohistochemistry; IF, immunofluorescence; IRS, immunoreactive score; OS, overall survival; PFS, progression free survival; PUFAs, polyunsaturated fatty acids; ROS, reactive oxygen species; TCGA, the Cancer Genome Atlas; TMZ, temozolomide; y, years.
